# Steroid anabolic and catabolic hormones in highly and recreationally trained female athletes across the menstrual cycle

**DOI:** 10.14814/phy2.70588

**Published:** 2025-09-29

**Authors:** Katia Collomp, Carole Castanier, Caroline Teulier, Agnès Olivier, Léane Blanchard, Juliette Bonnigal, Emmanuelle Duron, Eric Favory, Mathieu Zimmermann, Virgile Amiot, Valérie Carité, Marine Lefebvre, Cynthia Mongongu, Magnus Ericsson, Corinne Buisson

**Affiliations:** ^1^ CIAMS Université d'Orléans, Université Paris‐Saclay Orléans France; ^2^ LADF Université Paris‐Saclay Orsay France; ^3^ IFCE, Cadre Noir Saumur France; ^4^ Hôpital Brousse, AP‐HP, Villejuif, France‐ Equipe INSERM MOODS‐CESP Kremlin‐Bicêtre France; ^5^ Médecine du Sport CHU Orléans Orléans France; ^6^ Service de Santé Des Etudiants Université Paris‐Saclay Orsay France

**Keywords:** androstenedione, cortisol, estradiol, PANAS, testosterone, training status

## Abstract

Sex hormone fluctuations across the normal menstrual cycle (NMC) may affect women's performance and health, but literature is sparse on the estradiol (E2) impact on anabolic and catabolic hormones and well‐being in female athletes. We therefore measured in 21 NMC anaerobically trained female athletes: 12 highly (HT) and nine recreationally (RT) during late luteal/early follicular (Low E2) and late follicular/peri‐ovulatory (High E2) phases: DHEAS, DHEA, androstenedione (A4), testosterone (T), sex hormone‐binding globulin (SHBG), cortisol, free androgen index (FAI = T/SHBG), and their relative ratios. Body composition and well‐being, assessed by positive and negative affects (PANAS), were determined in parallel. In High versus Low E2, subjects showed a significant percentage increase in T, A4 (*p* < 0.001) and FAI (*p* < 0.05), with high A4/T correlation (*r* = 0.78), without any change in the other parameters. While no group difference was detected in hormone concentrations, HT versus RT subjects presented higher muscle mass and positive affects and lower FAI (*p* < 0.05). In conclusion, T and A4 were modulated across NMC, probably resulting from a variable ovarian contribution, but without inducing any change in the anabolic/catabolic balance, regardless of the physical training level. However, high anaerobic training may enhance T sensitivity in view of the FAI, body composition and well‐being outcomes.

## 1 INTRODUCTION

There is still no consensus at the present time on whether fluctuations in sex hormones during the normal menstrual cycle (NMC) may or may not significantly affect female athletes' performance and well‐being. Indeed, various experimental studies and reviews have reported significant changes in women throughout the menstrual cycle, the phase that seems to impact at least some women is the period close to menstruation, that is, the late luteal and early‐follicular phases, when estradiol (E2) concentrations are lowest (Carmichael et al., 2021; Findlay et al., 2020; de Janse Jonge, 2003; McNulty et al., 2020). Ovaries are the main source of E2, but it is also formed by aromatization of testosterone or synthesized by estrone's reduction by 17β‐hydroxysteroid dehydrogenase enzymes in tissues outside of the gonads. In women, estradiol is considered to have a negative relationship with muscle stiffness (Bell et al., 2012; Ham et al., 2020) and neuroexcitatory effects with greater quadriceps and handgrip strength (Sarwar et al., 1996) and voluntary activation (Ansdell et al., 2019). In parallel, high E2 levels are generally associated with a positive mood and high levels of well‐being and self‐esteem (Farage et al., 2008), while pre‐menstruation and menstruation are often linked to a negative mood, with possible direct connections to low E2 levels. Therefore, women experience a low level of negative affect during the end follicular phase, which increases a paroxysm at the time of menstruation (Bäckström et al., 1983; Sanders et al., 1983).

The major androgens in women, listed in descending order of serum concentration (Burger, 2002), include dehydroepiandrosterone sulfate (DHEAS), dehydroepiandrosterone (DHEA), androstenedione (A4), testosterone (T), and dihydrotestosterone (DHT), a peripheral product of T conversion that circulates in very low concentrations in serum. Whereas DHEAS is an adrenal product, regulated by the adrenocorticotropic hormone (ACTH) and acting as a precursor for the peripheral synthesis of more potent androgens, DHEA is produced by both the ovary and adrenal, as well as being derived from circulating DHEAS. A4 and T are products of the ovary and the adrenal, with 25% of T secreted by the adrenal zona fasciculata, 25% by the ovarian stroma, the remaining 50% being produced from circulating A4 (Abraham, 1974; Badawy et al., 2021; Burger, 2002). Approximately two‐thirds of the circulating T is bound and inactivated by sex hormone binding globulin (SHBG), while one‐third is bound to albumin. Free active circulating T, estimated by either free blood T concentrations, saliva concentrations, or the free androgen index (FAI), that is, total testosterone/SHBG, constitutes 1%–5% (Hirschberg, 2020; Pilz‐Burstein et al., 2010). Testosterone, the most potent androgen after DHT, is known to directly or indirectly stimulate different physiological mechanisms that are responsible for skeletal muscle gain and athletic performance improvement in men (Basualto‐Alarcon et al., 2013; Cardinale & Stone, 2006; Handelsman et al., 2018), with fewer works performed in women (Cook et al., 2012, 2018, 2021; Cook & Beaven, 2013; Crewther & Cook, 2018; Crewther et al., 2018; Hirschberg et al., 2020; Horwath et al., 2020). Although studies on hyperandrogenic women or with testosterone administration clearly demonstrated a link between testosterone and anaerobic performance (Hirschberg et al., 2020; Horwath et al., 2020; Notelovitz, 2002), there is still no consensus on the physical or psychological repercussions of physiological endogenous testosterone levels in non‐hyperandrogenic women, whose T concentrations are rarely studied. Indeed, some studies (Cook et al., 2012, 2018, 2021; Cook & Beaven, 2013; Crewther & Cook, 2018; Roli et al., 2018) have shown higher basal or exercise T concentrations in elite female athletes versus recreationally trained or non‐trained women, with load volume rather than load intensity considered the most important training variable for activating the endocrine system and stimulating muscle growth (Crewther et al., 2008). This increase in T was also considered by the authors to be not only a physiological but also a psychological performance factor via the behavioral mechanisms of motivation and attention (Cook & Beaven, 2013; Crewther et al., 2008). However, other studies have failed to establish a relationship between testosterone levels and training or performance (Ahmetov et al., 2020; Bezuglov et al., 2023; Muscella et al., 2022). Furthermore, combined hormonal contraception that lowers T levels did not alter physical performance (Crewther et al., 2018), while alterations in mood and sexual function in women yielded contradictory results (Burrows et al., 2012; Coelingh Bennink et al., 2017). Interestingly, some studies have shown increased blood T concentrations in healthy women around the time of ovulation (Abraham, 1974; Knutsson et al., 2023), while higher free saliva T concentrations were found in trained female athletes during this phase (Cook et al., 2018; Crewther & Cook, 2018). Nevertheless, potential variations across NMC in blood T together with all its androgenic precursors (i.e., DHEAS, DHEA, and A4) and SHBG concentrations in female athletes according to their training level have never been studied to our knowledge.

Last, numerous studies pointed out the role of cortisol (C) in physical performance with a dual role. Indeed, while cortisol has well‐known catabolic effects, cortisol secretion has been shown to be a necessary factor for physical performance with higher concentrations in trained subjects (Balthazar et al., 2012; Moyers & Hagger, 2023; Passelergue et al., 1995). However, there is still no consensus regarding the effects of menstrual cycle phase on cortisol responses (Altemus et al., 2001; Boisseau et al., 2013; Kraemer et al., 2013; Prado et al., 2025). In addition, while the testosterone/cortisol (T/C) ratio has long been used to estimate anabolic/catabolic balance and to predict either fatigue or underperformance in male athletes (Urhausen et al., 1995), literature in female athletes remains limited (Roli et al., 2018; Vervoorn et al., 1992), with no studies, to our knowledge, investigating the potential impact of the menstrual cycle phase on this T/C ratio or on the anabolic T precursors/C ratios.

The aim of the present study was therefore to determine whether NMC E2 fluctuations in female athletes impacted steroid anabolic (DHEAS, DHEA, A4, and T) and catabolic (C) hormone levels and their relative ratios, by comparing late luteal/early follicular phase (Low E2) and late follicular/peri‐ovulatory phase (High E2). In parallel, we investigated the influence of athlete physical training level and the possible link between hormone variations and well‐being, by assessing positive and negative affects.

## 2 MATERIALS AND METHODS

### 2.1 Participants

Twenty‐one female athletes, 12 highly trained athletes (Group 1: HT) and 9 recreationally trained students (Group 2: RT), mainly from the School of Sports of Paris‐Saclay, volunteered to participate in the study and signed a written consent form after being informed of the objectives and risks of the study. All procedures were approved by the ethics committee (ID‐RCB:2020‐A02965‐34) and were in accordance with the Declaration of Helsinki. Prior to the study, subjects were screened with a medical history and physical examination. Inclusion criteria required that HR participants be highly physically active training (6–11 times per week, 3–6 h per day, for at least 3 years), while RT participants were physically active for less than 8 h/week, that is, a training load representing less than one‐third of that of the HT group. Subjects in both groups performed various sporting disciplines that were not exclusively but mainly anaerobic, that is, judo, horse riding, climbing, sprinting, and shot put. Exclusion criteria were the presence of cardiovascular, liver, biliary or renal disease; hyperlipidaemia; high blood pressure; endocrinological disorder; oligomenorrhoea or amenorrhoea; hormonal contraception; premenstrual syndrome; pregnancy; history of thromboembolic disorder. Age (HT: 19.8 ± 1.1; RT: 19.8 ± 2.2 years), body mass (HT: 60.7 ± 8.1; RT: 58.5 ± 7.1 kg), and body mass index (HT: 22.5 ± 1.6; RT: 22.2 ± 1.8 kg/m^2^) were similar in both groups at the start of the study.

### 2.2 Study design

After the inclusion visit and after asking participants for their menstrual dates during the previous three cycles, NMC participants came to the laboratory during Low E2 (Day ‐2 to 4) and High E2 (Day 10 to 15) phases. Progesterone (PG) concentrations were determined to confirm that Day 10–15 corresponded to the late follicular/periovulatory phase and not to the luteal phase. The protocol for each trial was held at the same time of day to prevent diurnal variation. Participants were asked to maintain their normal food dietary intake during the experiment and to refrain from ingesting either caffeine or alcohol and strenuous efforts for at least 24 h prior to the trial sessions. The subjects reported no changes in their energy and fluid intake, exercise intensity, or training volume between the two sessions. With regard to medication, two subjects in the RT group took NSAIDs the day before the low E2 session, namely ibuprofen and flurbiprofen, the latter in combination with paracetamol and codeine. Regarding the mild menstrual pain reported by some participants, none of them were considered to be suffering from premenstrual syndrome.

### 2.3 Body composition

On trial days, subjects reported in a fasting state to the laboratory between 6:45 am and 7:45 am. After a 15 min rest, body composition was assessed by bioelectrical impedance analysis using a Tanita MC‐780 multifrequency segmental body composition analyzer, which included body mass, fat mass, muscle mass, and body water (total, intra‐ and extra‐cellular).

### 2.4 Blood analysis

Venous samples (6 mL) were then collected from an antecubital vein using standard procedures. Blood was immediately transferred into serum tubes and centrifuged at 3500 rpm for 10 min before serum was stored in a freezer at −20°C for later analysis. E2, PG, DHEAS, DHEA, C, and SHBG were determined by ELISA (kits from DRG Diagnostic®, i.e., EIA 2693, EIA 1561, EIA 1562, EIA 3415, EIA 1887, and EIA 2996, respectively), whereas T and A4 were determined by liquid chromatography coupled to mass spectrometry (LC–MS/MS, ThermoFisher Scientific®) following the World Anti‐Doping technical guideline (Laboratory guideline: Quantification of Endogenous Steroids in blood for the Athlete Biological Passport version 1.0 (2023)). Assays were made in duplicate for ELISA determination, and coefficients of variation for all parameters were always <10%.

### 2.5 Well‐being

Positive and negative affect schedule (PANAS) (Watson et al., 1988) is the most widely used psychological tool for assessing an individual's affective or emotional state in athletes. Positive affect refers to the extent to which an individual experiences positive emotions such as enthusiasm, determination, motivation, self‐confidence, and alertness, while negative affect measures the presence of negative emotions such as fear, anger, guilt, shame, and distress. By monitoring an athlete's positive and negative scores, it is possible to identify potential emotional barriers to performance and implement strategies to mitigate their impact. The PANAS, which assesses affect at a given moment in time, was then completed by the subjects 30 min after a standardized breakfast (400 kcal) in order to avoid any adverse effects related to fasting.

### 2.6 Statistical analyses

Results are presented as mean values ± standard deviation (SD). Firstly, unpaired *t*‐tests were used to check that there were no differences between the groups with regard to age and body weight. After evaluating the normality of the samples, differences between the trials were investigated: (1) To determine the impact of the E2 phase on parameters, we applied a 1‐factor analysis of variance (ANOVA or Kruskal–Wallis) with all subjects taken together; (2) To determine the impact of the E2 phase and training on parameters, we applied a 2‐factor (condition × group) ANOVA when normality was respected while Friedman repeated measures analysis of variance (condition) and Welch (group) being applied when non‐normal distribution of hormonal data. A post hoc Fisher test or pairwise comparison post hoc tests were then performed to determine which parameters showed significant differences in the event of analyses of variance revealing a significant main effect. Correlations were calculated using the Spearman rho correlation test. The null hypothesis was rejected at a *p* value <0.05.

## 3 RESULTS

### 3.1 Phase effects (Tables 1 and 2 and Figures 1, 2, 3)

**TABLE 1:** Endocrine and body composition parameters (mean ± SD) in highly trained (HT) and recreationally trained (RT) groups under Low E2 and High E2 conditions.

Parameters	HT group		RT group	
Mean SD	Low E2	High E2	Low E2	High E2
Endocrine				
E2 (pg/mL)	57.8 31.1	140.4 79.9	69.1 33.4	132.0 58.1
PG (ng/mL)	0.8 0.3	0.8 0.3	0.9 0.3	0.9 0.3
DHEAS (mg/L)	1.80 0.85	1.86 1.17	1.83 0.81	1.70 0.42
DHEA (ng/mL)	14.5 7.0	14.9 5.2	17.2 9.0	16.9 7.6
A4 (ng/mL)	1.36 0.50	1.76* 0.53	1.61 0.62	2.11* 0.73
T (ng/mL)	0.291 0.141	0.363* 0.143	0.306 0.115	0.402* 0.116
C (ng/mL)	202.5 89.9	215.4 87.6	200.1 54.3	239.4 58.2
Body composition				
Weight (kg)	61.4 7.7	60.9 8.7	58.8 7.2	58.4 7.5
Muscle mass (kg)	46.3 4.3	45.9 4.8	42.3+ 3.9	41.8+ 3.6
Fat mass (kg)	12.7 5.5	12.6 5.8	14.2 4.0	14.4 4.8
Fat mass (%)	20.1 6.5	20.1 6.7	23.9+ 3.9	24.2+ 4.7
Total water (kg)	33.8 3.4	33.6 3.6	32.1 3.0	31.7 2.7
Intracellular water (kg)	19.0 1.5	19.0 1.6	18.9 1.8	18.7 1.8
Extracellular water (kg)	14.0 1.2	13.9 1.4	13.2 1.2	13.0 1.2

* *p* < 0.05, significant phase difference.

^+^ *p* < 0.05, significant group difference.

**TABLE 2:** DHEA/A4, DHEA/T, A4/T, DHEA/C, and T/C ratios (mean ± SD) in highly trained (HT) and recreationally trained (RT) groups under Low E2 and High E2 conditions.

Parameters	HT group		RT group	
Mean SD	Low E2	High E2	Low E2	High E2
DHEA/A4	11.2 5.4	8.7 2.4	10.7 3.6	8.1 2.7
DHEA/T	58.3 37.7	43.5 12.8	62.2 38.4	44.1 23.1
A4/T	5.0 1.1	5.0 1.0	5.5 1.8	5.4 1.8
DHEA*1000/C	80.0 39.7	81.9 41.1	84.1 36.3	71.6 28.2
A4*1000/C	7.5 3.2	10.9 9.4	8.4 3.3	9.1 3.3
T*1000/C	1.6 0.8	2.2 1.7	1.6 0.6	1.8 0.6

**FIGURE 1:**
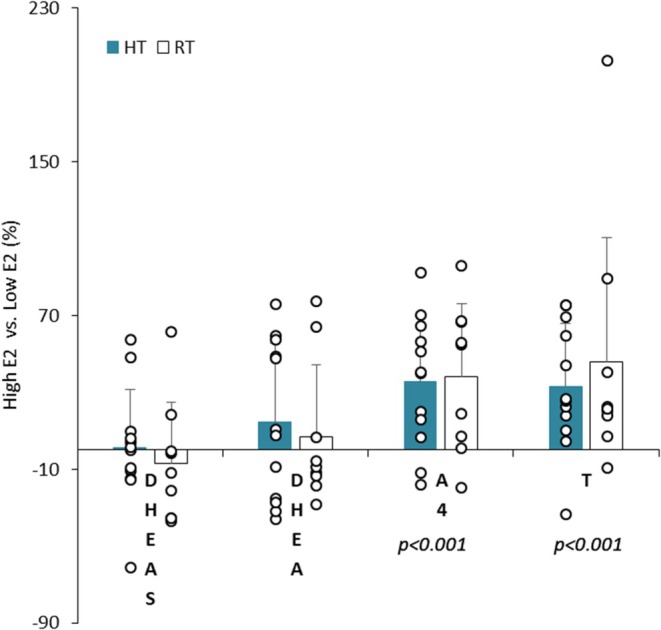
High E2 versus Low E2 anabolic hormone (i.e., DHEAS, DHEA, A4, and T) change expressed in percent (mean ± SD) in highly trained (HT) and recreationally trained (RT) groups.

**FIGURE 2:**
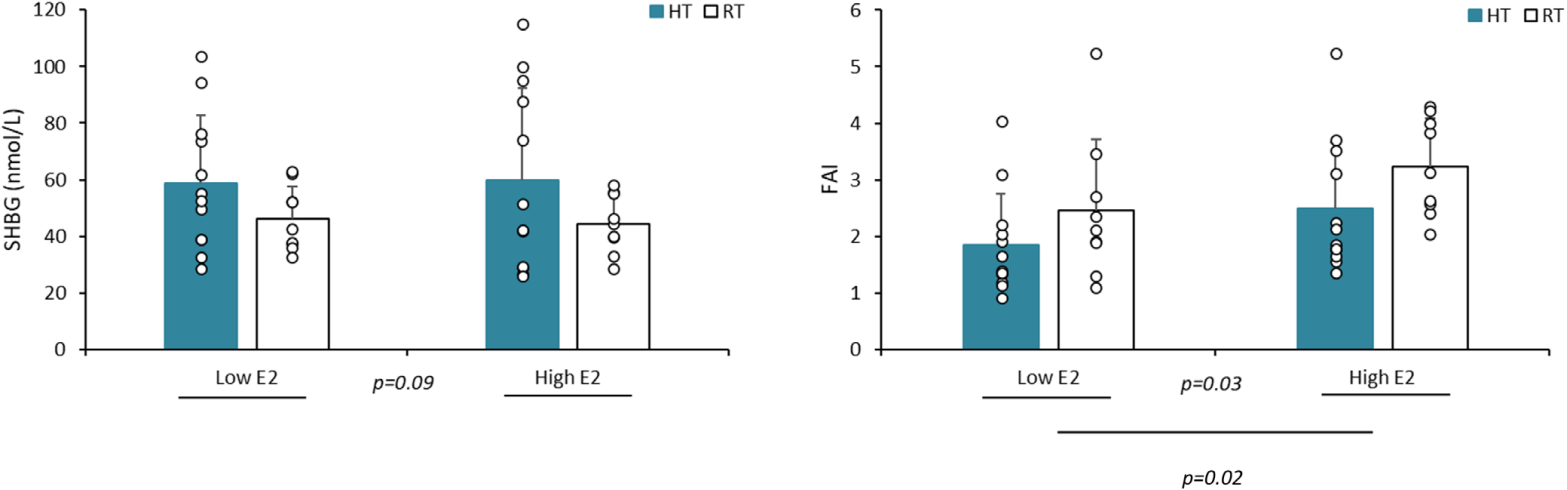
SHBG concentrations and free androgen index (FAI = testosterone*100/SHBG) (mean ± SD) in highly trained (HT) and recreationally trained (RT) groups under Low E2 and High E2 conditions.

**FIGURE 3:**
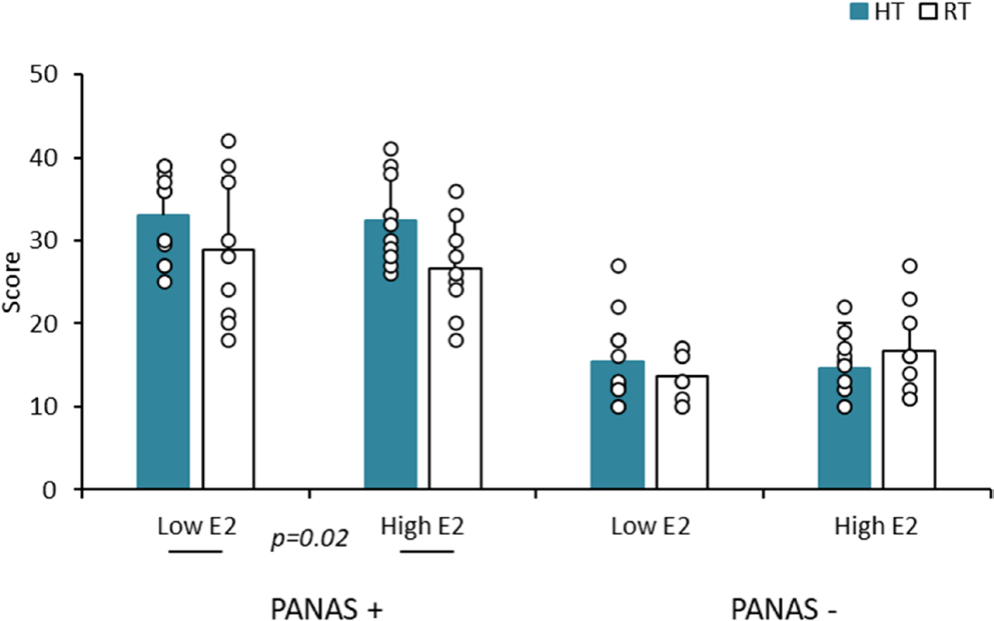
Positive and negative effects (mean ± SD) in highly trained (HT) and recreationally trained (RT) groups under High E2 and Low E2 conditions.

#### 3.1.1 Anabolic/catabolic hormones

In High E2 (136.8 ± 15.3 pg/mL) versus Low E2 phase (62.6 ± 6.9 pg/mL) (*p* < 0.001), when all subjects were taken together, with results expressed in both mean concentrations and %, there was a significant increase in T and A4 (*p* < 0.001) with a significantly higher FAI in High E2 versus Low E2 phase (*p* = 0.02) (Table 1, Figures 1 and 2). In contrast, no significant phase change was found in DHEAS, DHEA, and C or in the different hormonal ratios investigated (Table 2). Significant correlations were found between different hormonal parameters (Table 3) with a particularly strong correlation between A4 and T (*r* = 0.78, *p* < 0.01). In addition, there was a significant correlation between the % increase in E2, A4, and T in High versus Low E2 (E2 and A4: *r* = 0.43; E2 and T: *r* = 0.48; T and A4: *r* = 0.92, *p* < 0.01).

**TABLE 3:** Correlations (in bold where significant, *p* < 0.05) between the different hormonal parameters.

	E2	DHEAS	DHEA	A4	T	C	SHBG
E2	**1**	0.09	**0.35**	**0.36**	**0.31**	0.13	−0.07
DHEAS		**1**	**0.37**	0.12	0.13	0.17	0.22
DHEA			**1**	**0.62**	**0.43**	**0.34**	0.07
A4				**1**	**0.78**	0.25	0.04
T					**1**	**0.34**	**0.40**
C						**1**	**0.39**

#### 3.1.2 Body composition

No alteration in body composition was observed across NMC (Table 1).

#### 3.1.3 Well‐being

No change in positive or negative affect was found between High E2 and Low E2 (Figure 3).

### 3.2 Physical training level effects (Tables 1 and 2 and Figures 1, 2, 3)

#### 3.2.1 Anabolic/catabolic hormones

No significant difference between the two groups was found in the hormonal anabolic (DHEAS, DHEA, A4, and T) or catabolic (C) concentrations as well as on the hormonal ratio investigated (Table 1). However, HT subjects showed lower FAI (*p* = 0.03) than RT due to a trend towards higher SHBG (*p* = 0.09) in this group (Figure 2).

#### 3.2.2 Body composition

HT versus RT subjects exhibited higher muscle mass (kg and %) with consequently lower fat mass in % but not in kg (*p* < 0.05, Table 1).

#### 3.2.3 Well‐being

Positive affect was higher in HT versus RT (*p* = 0.02) while no change was detected for negative affect, regardless of the phase (Figure 3). A significant correlation was only found between positive affect and muscle mass (kg) (*r* = 0.36, *p* < 0.05).

## 4 DISCUSSION

There is little literature on the impact of E2 fluctuations on steroid anabolic and catabolic hormone concentrations and well‐being across NMC in female athletes. To our knowledge, this is the first study to provide such a robust hormonal profile, including testosterone and all its androgen precursors. Regardless of physical training level, we found a significant increase in some (T and A4) but not all (DHEAS and DHEA) androgenic anabolic hormones during High E2 versus Low E2, without any phase change in cortisol, anabolic/catabolic balance, or in the anthropometric and well‐being parameters studied. While no group difference was noted in anabolic or catabolic hormone concentrations, highly versus recreationally trained subjects had a lower free androgen index, combined with higher muscle mass and positive affect.

Abraham (1974) and Knutsson et al. (2023) reported in healthy active women a significant impact of the menstrual cycle on both blood T and A4 concentrations, with higher levels around ovulation, but no change in A4/T ratio or in DHEA levels that remained stable across NMC. In addition, Cook et al. (2018) observed the same menstrual cycle changes in saliva T, with a more marked effect in high‐performing female athletes with higher T concentrations. Partially according to these previous data, we found, irrespective of the subjects' physical training level, a change in mean T and A4 concentrations, with a significant % increase in T and A4, but not in DHEAS and DHEA, during High E2 compared with Low E2, with E2 levels within the normal range. In addition, given that SHBG concentrations remained similar throughout NMC, we consequently observed a significantly higher free androgen index in High versus Low E2 phases. In view of the positive strong correlation between A4 and T (*r* = 0.78), and as previously proposed by Abraham (1974) in normally menstruating nontrained subjects, it can be suggested that the ovarian contribution of T and A4 fluctuated across NMC, with a maximum contribution at mid‐cycle when E2 levels are high, probably resulting from the luteinizing hormone (LH) stimulation from the pituitary gland on the ovarian tissue, the absence of changes in DHEAS and DHEA being related to their unique or primary adrenal origin, respectively. Indeed, in parallel, we found similar cortisol levels during Low E2 and High E2 phases. It thus seems, according to most studies performed in women (Altemus et al., 2001; Boisseau et al., 2013; Kraemer et al., 2013), that basal cortisol concentrations in highly and recreationally trained female athletes were not subjected to the menstrual cycle, at least in athletes performing anaerobic sports disciplines. Furthermore, it is important to note that the phase changes in either A4 or T did not lead to any alteration in the anabolic/catabolic balance across NMC, given the lack of significant change in T/C or in the T precursors/C ratios studied. However, further studies appeared necessary to clarify the potential performance and well‐being repercussions of lower FAI in the Low E2 phase.

Our highly trained female athletes exhibited higher muscle mass than their recreationally trained counterparts, as classically reported in the literature (Karatrantou et al., 2017; Kraemer et al., 2004; Mero et al., 2013). However, contrary to previous studies (Cook et al., 2012, 2018, 2021; Cook & Beaven, 2013; Crewther & Cook, 2018; Roli et al., 2018), no difference between the two groups was found in anabolic and catabolic hormone levels, anabolic metabolism, or anabolic/catabolic balance in view of the ratios, irrespective of the NMC phase.

The absence of variation in cortisol concentrations between the two groups can be explained by the mainly anaerobic characteristics of the sports practised by the participants, as aerobic training appears to be a more powerful stimulator of the hypothalamic–pituitary–adrenal axis than anaerobic training (Athanasiou et al., 2023). However, we observed a decrease in FAI among our highly versus recreationally trained subjects, linked to a trend towards higher SHBG, that appears counterintuitive at first view. Indeed, some studies reported in female athletes practising anaerobic and mixed physical activities, without menstrual status taken into account, a positive correlation between free and total T levels and physical performance, with both baseline levels correlated with oxygen uptake and work capacity (Cook et al., 2012, 2018, 2021; Cook & Beaven, 2013; Crewther & Cook, 2018). On the contrary, no relationship between T levels and training or performance was established in other studies (Ahmetov et al., 2020; Bezuglov et al., 2023; Muscella et al., 2022). To our knowledge, SHBG and FAI were never investigated in female athletes regarding their physical training practices, but it is recognized that SHBG production depends on multifactorial regulation, with hormonal, metabolic, nutritional, and medicinal factors influencing SHBG levels (Bonnet et al., 2009; Knutsson et al., 2023; Toscano et al., 1992). As none of our participants had high levels of visceral fat, with similar BMI and absolute fat mass (in kg) between the two groups, it is unlikely that body fat levels played a role in our SHBG and FAI outcomes. Different works performed on other populations (elderly people, diabetics) illustrated increased SHBG concentrations with physical training (Hayes et al., 2015; Kim et al., 2017), while Maynar et al. (2010) showed an increase in SHBG in young male weightlifters after 20 weeks of strength training without any change in T, resulting in a decrease in FAI. The authors suggested that this decrease in FAI could reflect an increased demand and utilization of total T at the muscular level, with an increase in SHBG levels as a compensatory mechanism to decrease the urinary elimination of androgenic hormones (Maynar et al., 2010). Another hypothesis may be that highly anaerobic training leads to greater T sensitivity, without requiring an increase in the fraction of bioavailable testosterone. Indeed, we observed significant higher muscle mass, expressed both in kg and %, in HT compared with RT athletes. Furthermore, a more pronounced positive affect, correlated to muscle mass, was seen in HT versus RT athletes. Other factors that may influence the higher PANAS+ score in HT athletes remain to be determined, but based on the results of this study, we could exclude the roles of both cortisol as a stress factor and hydration level, as these did not differ between the two groups. It is interesting to note that both positive and negative affects remained stable across these two phases in both recreationally and highly trained subjects, contrary to what was found in untrained women (Bäckström et al., 1983; Sanders et al., 1983). It may therefore be suggested that female athletes at least without premenstrual syndrome would be less sensitive to estradiol fluctuations compared to their untrained counterparts, with further work needed to confirm and correctly interpret these last results.

This study had several limitations, including self‐reported information on diet, training, and medication, as well as the limited number of participants. In addition, we investigated the impact of E2 on anabolic and catabolic steroid hormones in female athletes, but unfortunately, it was not possible to establish a direct cause‐and‐effect relationship. Finally, the use of NAIDS and opiates by two participants during the low E2 phase may have influenced the PANAS score. We acknowledge these shortcomings but also highlight the strengths of the present study. All of the participants were highly motivated and provided accurate information, with full compliance to the protocol. To our knowledge, this was the first study to examine the relationship between E2 alone (without PG interaction) and the entire range of anabolic steroid hormones in female athletes, taking into account their level of physical training. Furthermore, given the growing concerns about the influence of sex steroids on behavior, mental health, stress resistance, and vulnerability in women, we have incorporated our subjects' perceived well‐being as a psychological component, making it an innovative and integrative pilot study in this field of research.

In conclusion, A4, T, and FAI appeared significantly modulated across NMC in both highly and recreationally trained athletes, with higher values in High E2 versus Low E2, probably due to a greater ovarian contribution during the first phase. However, these variations did not induce any change in the anabolic/catabolic balance. The level of anaerobic training did not appear to alter any steroid anabolic and catabolic hormone concentrations but may enhance T sensitivity in view of the FAI, body composition, and well‐being outcomes. Further studies are necessary to determine the mechanisms involved as well as how intense aerobic training, which is known to more significantly stimulate the hypothalamic–pituitary–adrenal axis, could affect anabolic steroids and steroid hormones across NMC.

## AUTHOR CONTRIBUTIONS

KC and CB were involved in study conception and design, data analyses, interpretation, and manuscript writing. CC, CT, and AO were involved in study conception and design, data analyses, and interpretation. LB, JB, and CM were involved in data analyses. EF, MZ, VA, VC, and ML were involved in participation in the experiment. ME and ED were involved in edition and revision of the manuscript for critical intellectual content. All authors read and approved the final version of the manuscript.

## FUNDING INFORMATION

This project was supported by the World Anti‐Doping Agency (WADA), the French Anti‐Doping Agency (AFLD), and the Institut Français du Cheval et de l'Equitation (IFCE).

## CONFLICT OF INTEREST STATEMENT

The authors declare no potential conflicts of interest that might be relevant to the contents of this manuscript.

## ETHICS STATEMENT

The study was approved by the ethics committee (ID‐RCB:2020‐A02965‐34) and was in accordance with the Declaration of Helsinki. Written informed consent was obtained from all participants after being informed of the objectives and risks of the study.

## Data Availability

Data are accessible upon reasonable request to the authors.
